# Bottlenose dolphins (*Tursiops truncatus*) aggressive behavior towards other cetacean species in the western Mediterranean

**DOI:** 10.1038/s41598-021-00867-6

**Published:** 2021-11-03

**Authors:** J. L. Crespo-Picazo, C. Rubio-Guerri, M. A. Jiménez, F. J. Aznar, V. Marco-Cabedo, M. Melero, J. M. Sánchez-Vizcaíno, P. Gozalbes, D. García-Párraga

**Affiliations:** 1 Fundación Oceanogràfic de la Comunitat Valenciana, Ciudad de las Artes y las Ciencias, C/Eduardo Primo Yúfera, 1B, 46023 Valencia, Spain; 2 VISAVET Center and Animal Health Department, Veterinary School, Complutense University of Madrid, Avda. Puerta del Hierro s/n, 28040 Madrid, Spain; 3 Marine Zoology Unit, Cavanilles Institute of Biodiversity and Evolutionary Biology, Science Park, University of Valencia, 46071 Valencia, Spain; 4 Departamento de Farmacia, Facultad Ciencias de la Salud, Universidad UCH CEU, 46115 Valencia, Spain

**Keywords:** Animal behaviour, Ecology, Behavioural ecology

## Abstract

Aggressive behavior of bottlenose dolphins (*Tursiops truncatus*) towards conspecifics is widely described, but they have also often been reported attacking and killing harbour porpoises (*Phocoena phocoena*) around the world. However, very few reports exist of aggressive interactions between bottlenose dolphins and other cetacean species. Here, we provide the first evidence that bottlenose dolphins in the western Mediterranean exhibit aggressive behavior towards both striped dolphins (*Stenella coeruleoalba*) and Risso’s dolphins (*Grampus griseus*). Necropsies and visual examination of stranded striped (14) and Risso’s (2) dolphins showed numerous lesions (external rake marks and different bone fractures or internal organ damage by blunt trauma). Indicatively, these lessons matched the inter-tooth distance and features of bottlenose dolphins. In all instances, these traumatic interactions were presumed to be the leading cause of the death. We discuss how habitat changes, dietary shifts, and/or human colonization of marine areas may be promoting these interactions.

## Introduction

Many cetaceans form inter-species groups that could benefit in foraging strategies^1^, though interspecific interaction in mixed groups could be complex to interpret^2^, leading even to the birth of hybrids on rare occasions^3,4^. However, close encounters between species groups can also lead to aggressive interactions between individuals^5–10^. An often-reported example of aggressive interactions between cetaceans are the attacks on harbour porpoises (*Phocoena phocoena*) by bottlenose dolphins (*Tursiops truncatus*). Such incidents of aggression are reported relatively frequently from UK and western USA waters and occasionally Northwest Spain, and may often lead to mortalities^11–17^. Barnet et al.^39^ also reported that aggression from bottlenose dolphins might have led to the death of at least one Risso’s dolphin (*Grampus griseus*), one long-finned pilot whale (*Globicephala melas*), one striped dolphin (*Stenella coeruleoalba*), and four short-beaked common dolphins (*Delphinus delphis*) on the southwest coast of England. More recently, an interspecific kill of a common dolphin by bottlenose dolphins was filmed and analyzed in detail in Northwest Spain^18^.

Intraspecific aggression is commonly observed in bottlenose dolphin behavior repertories in many different contexts such as male competition, dominance, or female access for copulation, supported by wide field data^19–22^. However, many hypotheses have been proposed to explain the causal factors behind the aggressive interactions between bottlenose dolphins and other cetacean species, which have been reviewed by several authors^13,14,18,23^. Aberrant behavior has been proposed, referred as the pattern that is outside the usual behavior for the species. Alternatively, it has been suggested that these interactions may result from bottlenose dolphins practice-fighting with the small porpoises^23^, or as a practice oriented to the acquisition of skills used in infanticidal attacks^13,18,23^. Other options such as high testosterone levels or sexual frustration have been considered when assessing the possible reasons driving interspecific traumatic interactions^23^. One usually discarded hypothesis is that dolphins are attempting to predate on the smaller cetaceans, although in most cases there is no evidence of attempted feeding^23^. However, perhaps the most feasible explanation is that it may be influenced by competition for resources^23^.

In this regard, many cetacean species adjust their distributions patterns in response to variability in food availability^24^. In the open ocean, it is generally accepted that cetaceans rarely display territoriality due to the lack of spatially-defined environmental features that may promote individuals or groups to demarcate their territories and patrol or defend them^25,26^. However, territoriality has been hypothesized in resident bottlenose dolphins from Scotland excluding visitors from productive deep waters during winter^26^. On that way, a potential enabler of predictable resources could be fish farms. These facilities often lead to increases fish biomass in the vicinity of the cages^27^. In fact, this increase in prey availability induces a greater presence of fish predators, including dolphins. Multiple reports, relate bottlenose dolphins aggregating around aquaculture facilities^28–33^, including the present study area where bottlenose presence around fish farms has been reported in greater numbers and all year around compared to other areas in the same study^34^. If food patches created by aquaculture increase site-fidelity in coastal dolphins, then some individuals or groups could aggressively defend these spatially-fixed resources^27^. Although the striped dolphin is mainly pelagic, it has also been suggested that, in the Mediterranean, the species is shifting from oceanic to neritic habitats due to prey increases in coastal waters^35^.

Here, we report on necropsies from fourteen striped dolphins and two Risso’s dolphins that stranded at different spots along the Valencian Community shoreline. In all instances, aggressive interactions with bottlenose dolphins were proposed as the leading cause of death.

## Results

We conducted necropsies on 136 dolphins along the 11 years of study, including 13 bottlenose dolphins, 7 short-beaked common dolphins, 2 long-finned pilot whales, 9 Risso’s dolphins and 105 striped dolphins. Of the 105 striped dolphins stranded in the study area, 17 (16%) stranded in the province of Castelló, 29 (28%) in València and 59 (56%) in Alacant. We observed on 14 of the striped dolphins, including eight males and six females, both macroscopic and microscopic compatible lesions, external examination (14/14) and internal (13/14), with bottlenose dolphins’ traumatic interactions that were likely the leading cause of death. Seven striped dolphins stranded in València province and the other 7 in Alacant (24% and 12% of performed necropsies in the area). No cases with similar injuries were found in the province of Castelló. On the basis of growth rates and total lengths published for striped dolphins in the western Mediterranean^36,37^ and state of gonadal development on internal examination, we estimated that of the affected dolphins, 6 were calves (considering until weaning), 3 were juveniles and 5 were sexually mature. Similar lesions were also present on two live stranded female Risso’s dolphins that died within 10 days of rehabilitation associated to infection, traumatic injuries and severe hypovolemic shock. One dolphin stranded in Valencia and the other in Alacant. One of these two dolphins was a calf and the other was a juvenile close to sexual maturity^38^.

**Figure 1:**
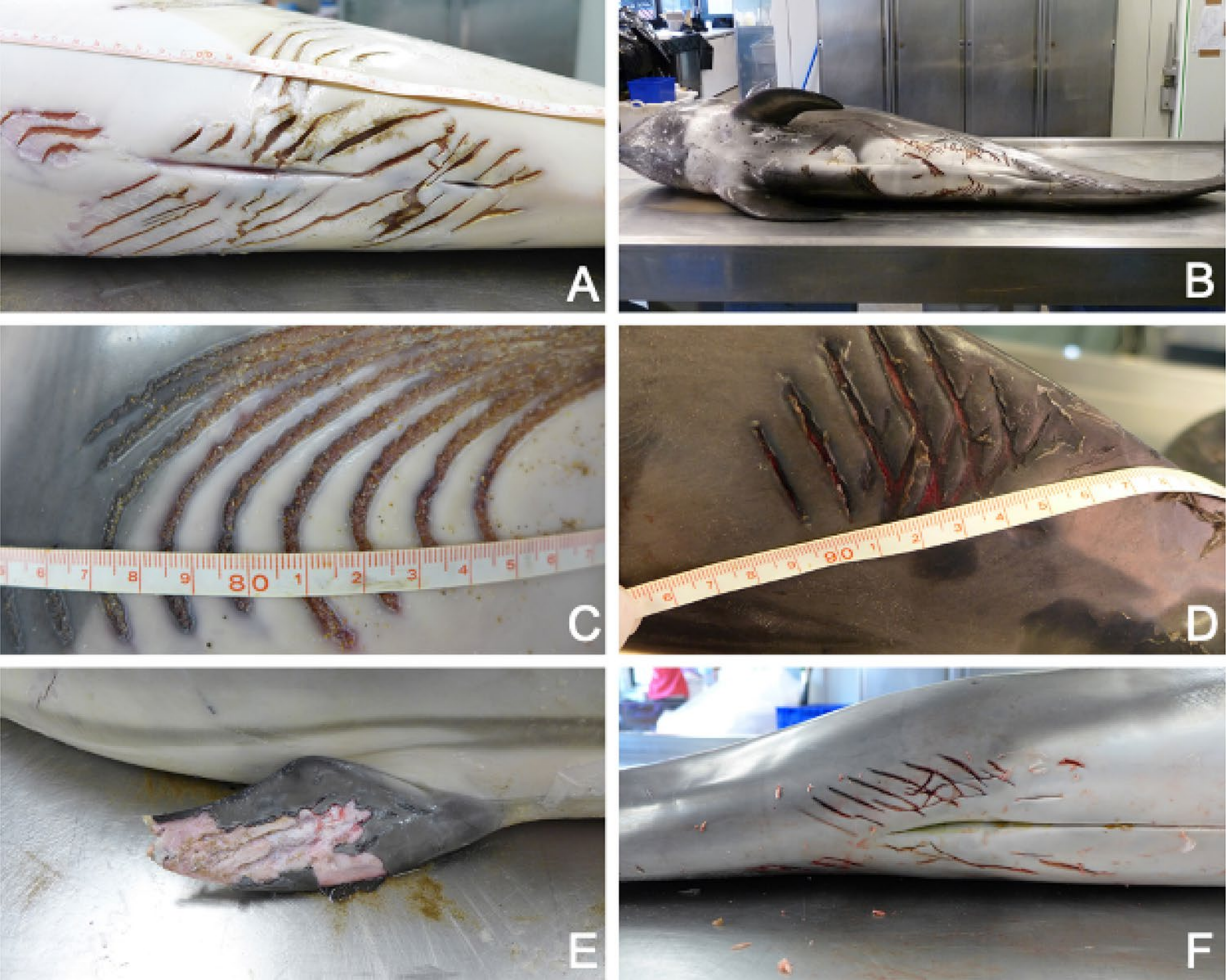
Main external lesions. Representative rake marks over different anatomical regions. (**A**) Male striped dolphin (Sc17.03.15), severe presence of parallel incisions, from 0.5 to 1.5 cm deep, in the perigenital area. (**B**) Female Risso’s dolphin (Gg29.03.13), multifocal presence of rake marks ventrally along the animal. (**C**) Male striped dolphin (Sc01.11.15), detail of the rake marks caudally in the left flank. (**D**) Female striped dolphin (Sc01.04.17), appearance of the rake marks laterally on the peduncle. (**E**) Male striped dolphin (Sc17.03.15) showing rake marks on the pectoral fin and loss of skin layer in the affected area. (**F**) Rake marks in the genital area in the second Risso’s dolphin case (Gg28.05.17).

**Figure 2:**
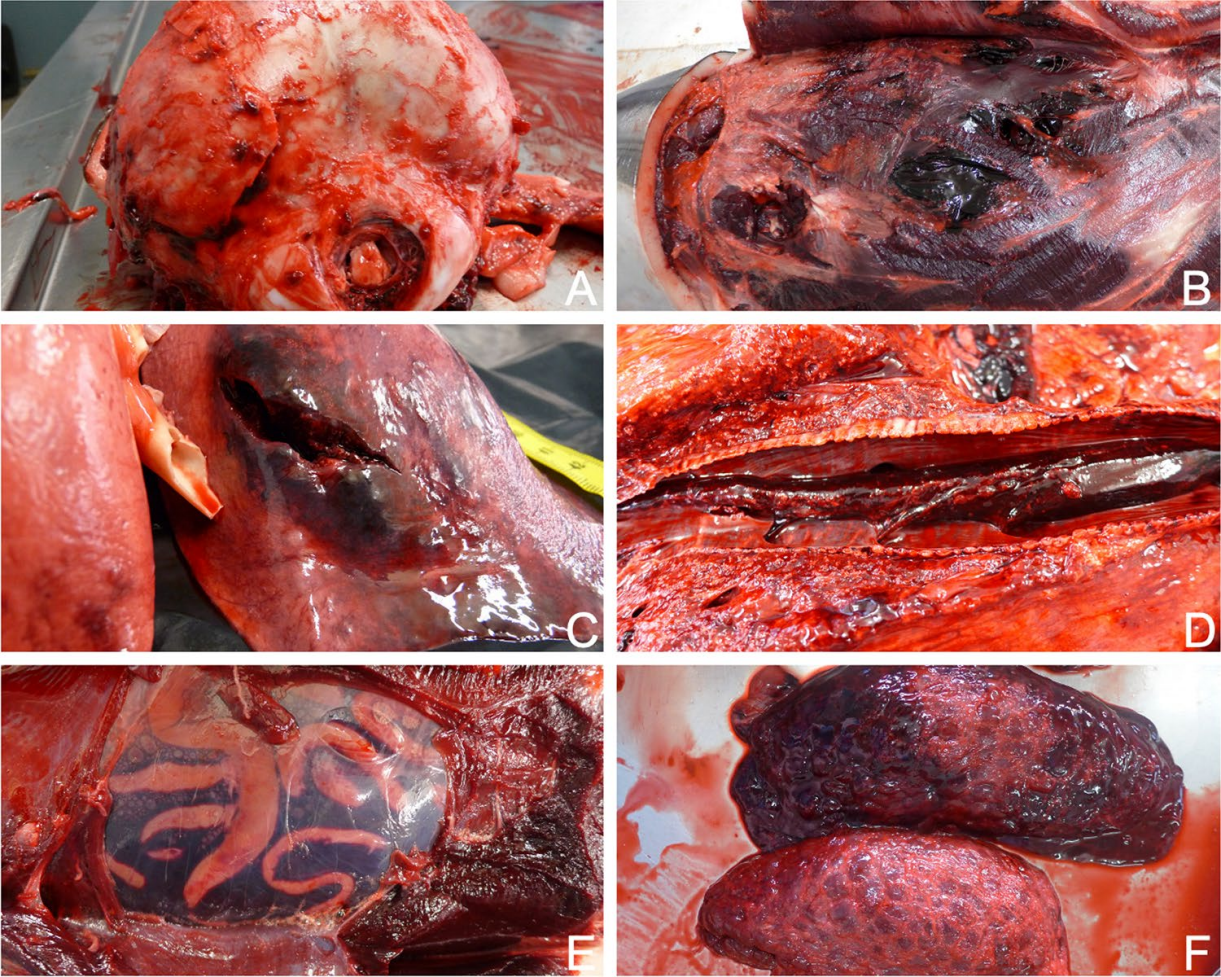
Representative internal macroscopic findings in striped dolphins. (**A**) Skull fracture (Sc30.03.11). (**B**) Multiple intercostal muscle fracture, hematoma and hemorrhage (Sc01.11.15). (**C**) Lung fracture and hematoma (Sc30.03.11). (**D**) Large intrabronchial clot (Sc18.02.15). (**E**) Hemoabdomen (Sc12.01.16). (**F**) Enlarged kidney, severe subcapsular hemorrhage (Sc01.04.17).

All external lesions on both striped and Risso’s dolphins consisted of three to fifteen parallel fresh lacerations (rake marks) that were spaced 1 to 1.2 cm apart (Fig. 1). The location of the skin lacerations varied between individuals. Of the 16 dolphins with these lacerations, 12 presented them on the fluke, 11 had them around the genital area, and 11 had them around the peduncle. Another four individuals also showed lacerations on their dorsal and pectoral fins, flank, and blowhole (Fig. 1). Internal macroscopic examination revealed severe hematomas and hemorrhages in the subcutaneous space and muscles; bone lesions including ribs, skull and spine fractures; hemothorax with extensive lung lacerations and pneumothorax; hemoabdomen secondary to liver ruptures, and even subcapsular hemorrhages in kidneys (Fig. 2). Moderate to severe leptomeningeal congestion was found in nine individuals and a cerebral hemorrhage was observed in a single individual that had suffered from a cranial fracture. In all cases acute and subacute lesions were microscopically observed including hemorrhage, oedema and erythrophagocytosis. Mild meningoencephalitis was found in two individuals. All main lesions detected were consistent with the occurrence of a traumatic event. Given the severity of the internal injuries, by extension and/or by affected organs, they were considered the cause of death.

One dolphin tested positive for Cetacean morbillivirus (CeMV) but, according to histopathology of tonsils and lung tissues, it was at an initial infection stage. No individuals tested positive for *Brucella* spp. or herpesvirus through PCRs.

More details on the size, gender, stranding date, stranding location and main pathological findings are presented in Tables 1 and 2.

**Table 1:** Summary information for 16 dolphins that stranded on the coastline of the Valencian Community, Spain and were suspected to have suffered from inter-specific interactions with bottlenose dolphins.

Case	ID	Species	Stranding date	Stranding location	Gender	Size (cm)
1	Sc30.03.11	*Stenella coeruleoalba*	30th March 2011	Altea	Male	123
2	Gg29.03.13	*Grampus griseus*	29th March 2013	València	Female	245
3	Sc27.09.13	*Stenella coeruleoalba*	27th September 2013	Elx	Male	174
4	Sc12.12.13	*Stenella coeruleoalba*	12th December 2013	Elx	Female	205
5	Sc18.02.15	*Stenella coeruleoalba*	18th February 2015	Sueca	Female	192
6	Sc14.03.15	*Stenella coeruleoalba*	14th March 2015	València	Male	177
7	Sc17.03.15	*Stenella coeruleoalba*	17th March 2015	València	Male	133
8	Sc01.11.15	*Stenella coeruleoalba*	1st November 2015	Sueca	Male	180
9	Sc10.10.16	*Stenella coeruleoalba*	10th October 2016	València	Male	155
10	Sc01.12.16	*Stenella coeruleoalba*	1st December 2016	El Saler	Female	103
11	Sc20.02.17	*Stenella coeruleoalba*	20th February 2017	Torrevieja	Male	200
12	Sc01.04.17	*Stenella coeruleoalba*	1st April 2017	Dénia	Female	198
13	Gg28.05.17	*Grampus griseus*	29th May 2017	Torrevieja	Female	162
14	Sc29.12.19	*Stenella coeruleoalba*	29th December 2019	Benidorm	Male	212
15	Sc22.05.20	*Stenella coeruleoalba*	22th May 2020	Torrevieja	Female	115
16	Sc15.10.20	*Stenella coeruleoalba*	15th October 2021	Gandía	Female	95

Size (total length) was measured from tip of upper jaw to fluke notch.

**Table 2:** Injuries observed on all 16 dolphins that had evidence of aggressive interactions with bottlenose dolphins. Severity was indicated as follows: (+) mild, (++) moderate, (+++) severe.

Case id	Rake marks	Haematoma	Haemo-thorax	Pneumo-thorax	Haemo-abdomen	Broken bones	Gross tissue lessions	Microscopic tissue lessions	Gastric content
1. Sc30.03.11	+	++	+	+		+	++	+	
2. Gg29.03.13	+++	+			+			++	
3. Sc27.09.13	++	+					+	+	+
4. Sc12.12.13	+	+	+++	+			+	++	++
5. Sc18.02.15	+	++	+++	+		+	++	+	++
6. Sc14.03.15	+	++					+	+++	+++
7. Sc17.03.15	+++	++	+++		+++		+++	+++	
8. Sc01.11.15	+	++	+++	+	++		+++	+++	+
9.Sc10.10.16	++	++	+++	+	++	++	+++	++	+
10. Sc01.12.16	+	+	+		+++	+	+++	++	+
11. Sc20.02.17	+	+	++	+			+	++	+++
12. Sc01.04.17	+	++	+++	+	+++		+++	++	+++
13. Gg28.05.17	+	+			+			+	++
14. Sc29.12.19	+	++	+++	+			+++	++	++
15. Sc22.05.20	+++	−	−	−	−	−	−	−	−
16. Sc15.10.20	++	+			++		++	−	

Empty box denotes no lesion and (**–**) no data available. In pneumothorax column ( +) denotes presence and empty box absence. Note: gastric content was not representative in case 2 (Gg29.03.13) as it was kept under rehabilitation for 10 days. Case 9 (Sc1.12.16) gastric content was compatible with milk. Case 15 was not internally explored.

## Discussion

We provide evidence from necropsies that suggests that aggressive interactions from bottlenose dolphins led to the death and/or stranding of 14 striped dolphins and two Risso’s dolphins in the western Mediterranean. Supporting this claim, rake marks and lesions that consisted of a series of parallel lacerations that were spaced 1 to 1.2 cm apart were present. These distinctive lesions were likely to have been caused by the teeth of an odontocete and moreover, spacing between the lacerations only matches the inter-dental distance for a single odontocete species in the western Mediterranean—the bottlenose dolphin^39^. Wounds are considered to be shark-inflicted if they are crescent-shaped, consistent with a shark's jaw. Lesions inflicted by sharks are usually deep, wide-spaced lacerations, which may be coupled with punctures of individual teeth^40^. Furthermore, many individuals also exhibited evidence of blunt-force trauma to the internal organs that may be attributable to ramming and tail slapping. Such aggressive behavior in bottlenose dolphins towards other cetacean species has been previously reported in other regions (particularly in UK, the western USA waters and Northwest Spain)^12–14,18,23,39^; however, this behavior is commonly focused towards conspecifics and harbor porpoises^5–8,10,13,14,16,23,39,41–44^. Other possible causes of traumatic death were ruled out in the striped dolphins in the absence of other external and internal lesions compatible with fisheries interactions, vessel collisions or live stranding^9,45–47^. Here, we report on the first evidence of bottlenose dolphins aggressing striped and Risso’s dolphins in the Mediterranean Sea.

**Figure 3:**
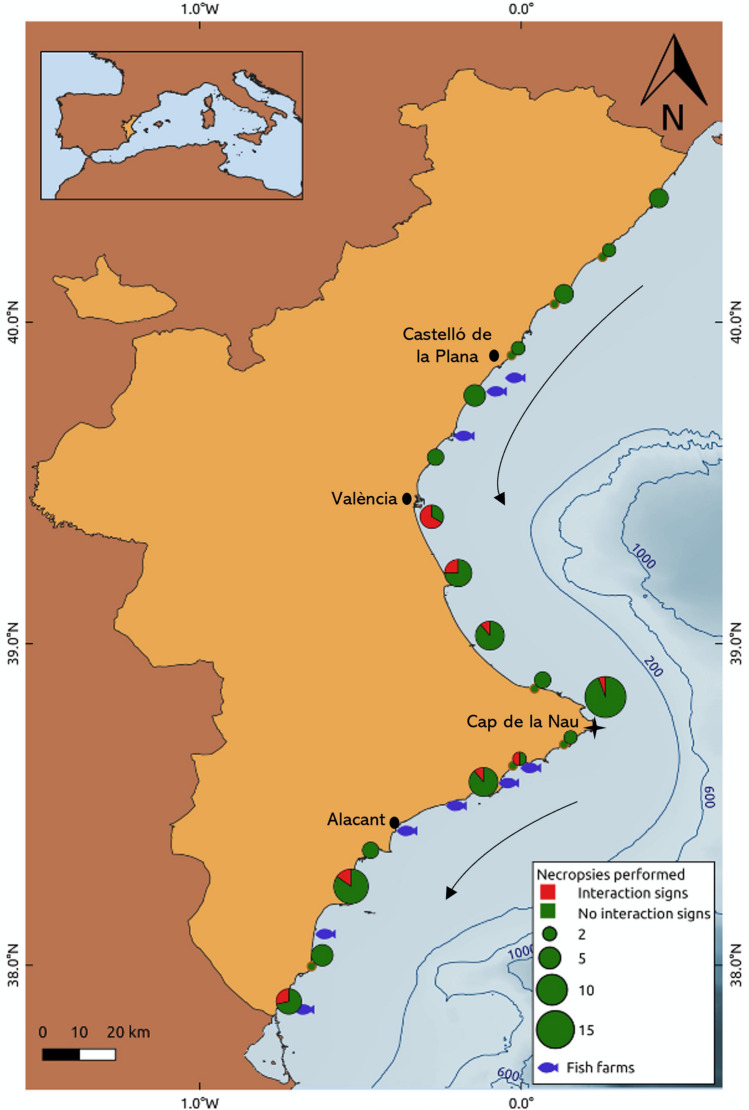
Stranding cases and fish farm locations. Necropsies performed every 25 km on well preserved striped dolphins (n = 105) during the study period. Individuals dead by traumatic interaction are represented in red (right). Black arrows show main sea superficial currents.

Coastal waters of the Valencian Community contain an estimated 15,778 striped dolphins, 1333 bottlenose dolphins, and 493 Risso’s dolphins, with all species being present all year round^48^. Each species generally inhabits different habitats based on depth^49,50^. Specifically, bottlenose dolphin primarily inhabits waters between 0 and 1000 m, striped dolphins between 900 and 1900 m, and Risso’s dolphins between 1500 and 2500 m. Nevertheless, the ranges of these species do still periodically overlap. For example, striped dolphins in the western Mediterranean regularly move over the continental shelf to hunt for neritic prey^35^ and similar behavior for this species has also been reported in the Ligurian Sea^51^. Bottlenose dolphins have also been reported to occasionally move away from the continental shelf and into deep-waters to forage^49^. Such overlap in the habitats of these species opens the possibility for inter-specific interactions. It should also be noted that the strandings associated with inter-specific interactions where often recorded in areas where the continental shelf is narrower (Fig. 3), and thus in areas where bottlenose and striped dolphin habitats are relatively near to each other. In fact, Castelló and València have a very similar number of kilometers of coastline (139 versus 135 respectively) but a big difference in the length of the continental shelf, being considerably smaller in the province of València, where the percentage of stranded dolphins with signs of traumatic interaction is considerably higher than in Castelló (24% versus 0% respectively).

If there is overlap in the ranges of these two species, there is also potential for competition for food resources. While most diet studies of striped dolphins suggest that these species primarily prey on pelagic and/or bathypelagic cephalopods^52^, a recent study in the area also identified that these striped dolphins will frequently prey on oceanic and neritic prey items^35^. Furthermore, striped dolphins are opportunistic predators^53^ and, in recent years, could be spending more time foraging in neritic habitats as a response to local increases in neritic prey, such as juvenile hake (*Merluccius merluccius*) and southern short fin squid (*Illex coindetii*)^35^. This is also supported by evidence from boat surveys, which suggest that striped dolphin sightings over the continental shelf have increased in recent years^54^. Occasionally, sightings of striped dolphins have even been reported in the vicinity of aquaculture facilities^48^. This could likely bring the striped dolphins into direct competition with the bottlenose dolphins as their primary prey in the Mediterranean is hake^55^. As for Risso’s dolphins, this species predominantly feeds on squid at depth^56^.

One of the hypothesis to consider in the surely multifactorial explanation of this behavior is the possible influence of fish farms. Bottlenose dolphins have been reported to aggregate around aquaculture facilities, presumably to opportunistically feed on the local aggregations of wild fish around the structures^31,34,57^. Indeed, Bonizzoni et al.^29^ found a higher occurrence of bottlenose dolphin occurrence within 20 km around fish cages. Furthermore, while territoriality is not a commonly-observed behavior in cetaceans, it has been suggested to be possible in situations concerning localized and predictable food sources^25,26^ such as fish farms. Thus, the presence of fish farms could trigger territoriality behavior displayed in bottlenose dolphin against striped dolphin in coastal areas close to aquaculture facilities. Such behavior has already been proposed to be occurring based on a 5-year study of dolphin presence around fish farms in the western Mediterranean^27^. In support of this hypothesis, the present study revealed that 12 of the 16 strandings reported here occurred within a range of 25 km of the fish farms (Fig. 3).

We cannot rule out other hypotheses discussed in the literature by various authors^13,18,23^, either as a single factor or as a set of motivations triggering the traumatic interaction, although some behaviors such as infanticide have not been described in the Mediterranean. For some proposed reasons such as the development of fighting skills in bottlenose dolphins, high testosterone levels or sexual frustration^13,14,23^, or species overlap due to niche and habitat changes, may favor the development of this behavior.

A mix of factors seems to have contributed to facilitate interactions between bottlenose and striped dolphins, but these may not be the same in the case of the Risso’s dolphins. Indeed, both individuals stranded alive and exhibited poor body condition. These facts are indicators of a previous chronic or subacute process and could denote that other reasons facilitating dolphin encounter, even the minor injuries found could indicate different attack/defense behavior from both implicated species. Incorporation of new cases if they occur in the future would allow deeper analyses. Bottlenose dolphins did not acutely kill those individuals, but their injuries contributed to the stranding and final death.

Bottlenose dolphins appear to be an important cause of mortality in striped dolphins over the study years, comprising up to 27.5% (4/11) of the causes of death in striped dolphins necropsied during 2015. Stranding numbers likely represent only a small fraction of animals that actually die at sea, being strandings estimated to be one order of magnitude below the number of individuals dying at sea^58–60^. According to this, the mortality posed by bottlenose dolphins on striped dolphins in the western Mediterranean should be monitored to decipher its impact on local populations.

Finally, it is worth considering how inter-specific cetacean interactions could affect the risk of infectious disease transmission. Close contact from aggressive interactions would likely increase the possibility for infection via body fluids or tissues exposition (blood, urine, secretions…). Some infectious diseases could also simply be transmitted by close contact between individuals^61^ and CeMV and *Brucella ceti* are commonly-diagnosed infectious diseases for striped dolphins in the study area^62,63^. In fact, one of the animals in this study tested positive for CeMV.

## Conclusion

We provide evidence suggesting that aggressive interactions from bottlenose dolphins were the primary cause of death for 14 striped dolphins as well as involved in the stranding and death of two Risso’s dolphins. We suggest that a combination of shifting ranges and ecological overlap could have made interspecies interaction more likely. Territoriality around fish-farms has been hypothesized as a possible contributing factor. Nevertheless, it is necessary to further study this phenomenon to determine unequivocally which factors are driving such interactions. Specifically, additional research into how human-induced changes in marine ecosystems could affect cetacean behavior and their interactions with other species will improve our understanding of potential ecosystem effects.

## Material and methods

### Permits and study area

Post-mortems of all dolphins were conducted under a collaborative official agreement between the *Conselleria d'Agricultura, Desenvolupament Rural, Emergència Climàtica i Transició Ecològica* of the Valencian Government. Dolphins were encountered stranded along the coastline of the Valencian Community (518 km) in eastern coast of Spain (40°31′N–0°30′E to 37°50′N–0°45′W).

### Dolphin strandings

Post-mortems were performed, following standard protocols as outlined in Kuiken and Hartmann^64^, on any well preserved (Code 1 to 3)^65^ cetacean carcass encountered within the study area between January 2010 and October 2020. The external inspection included examination of eyes, mouth, blowhole, umbilicus, genital opening, anus, and skin. Scars, abscesses, ulcerations, erosions, and wounds were documented, making note of the size (length, width, and depth), shape, color, texture, location, and distribution. Internal tissues were systematically examined documenting any abnormality as detailed for external examination^66^. Photographic record was taken from normal external and internal structures and abnormal findings. External lesions compatible with rake marks from conspecifics or interspecific individuals were specifically registered and compared with reports from patterns of lesions reported on the literature^9,39,40,67^. Fresh tissue samples of brain, spinal cord, heart, lung, kidneys, liver, selected lymph nodes, tonsil, thymus, gastrointestinal tract, pancreas, spleen, urinary bladder, gonads, blubber, skeletal muscle, and skin were fixed in 10% neutral buffered formalin for histopathology, refrigerated for microbiology, and frozen for molecular diagnosis. Molecular diagnosis was also used to test for CeMV^68^ and herpesvirus^69^. Finally, serum samples and/or cerebrospinal fluid were collected from live and dead strandings respectively to test for brucellosis using the standard Rose Bengal agglutination test^70^.

Map was generated with QGIS 3.10.4-A Coruña (https://qgis.org/es/site/forusers/download.html). Fish farm data was obtained from public repositories on request to local authorities (https://agroambient.gva.es/es/web/pesca/acuicultura).

## Data Availability

All data are available on reasoned request to the corresponding author.
